# Outcome of left heart mechanical valve replacement in West African children - A 15-year retrospective study

**DOI:** 10.1186/1749-8090-6-57

**Published:** 2011-04-19

**Authors:** Frank Edwin, Ernest Aniteye, Mark Mawutor Tettey, Martin Tamatey, Kwabena Frimpong-Boateng

**Affiliations:** 1 National Cardiothoracic Center, Korle Bu Teaching Hospital, P. O. Box KB 846, Accra, Ghana

**Keywords:** mechanical valve replacement, rheumatic heart disease, mitral valve, aortic valve, anticoagulation, West Africa, children

## Abstract

**Background:**

The West African sub-region has poor health infrastructure. Mechanical valve replacement in children from such regions raises important postoperative concerns; among these, valve-related morbidity and complications of lifelong anticoagulation are foremost. Little is known about the long-term outcome of mechanical valve replacement in West Africa. We sought to determine the outcome of mechanical valve replacement of the left heart in children from this sub-region.

**Method:**

We conducted a retrospective review of all consecutive left heart valve replacements in children (< 18 years old) from January 1993 - December 2008. The study end-points were mortality, valve-related morbidity, and reoperation.

**Results:**

One hundred and fourteen patients underwent mitral valve replacement (MVR), aortic valve replacement (AVR) or mitral and aortic valve replacements (MAVR). Their ages ranged from 6-18 years (13.3 ± 3.1 years). All patients were in NYHA class III or IV. Median follow up was 9.1 years. MVR was performed in 91 (79.8%) patients, AVR in 13 (11.4%) and MAVR in 10 (8.8%) patients. Tricuspid valve repair was performed concomitantly in 45 (39.5%) patients.

There were 6 (5.3%) early deaths and 6 (5.3%) late deaths. Preoperative left ventricular dysfunction (ejection fraction < 45%) was the most important factor contributing to both early and late mortality. Actuarial survival at 1 and 15 years were 98.1% and 94.0% respectively. Prosthetic valve thrombosis occurred in 5 patients at 0.56% per patient-year. There was 1(0.9%) each of major bleeding event and prosthetic valve endocarditis. Two reoperations were performed at 0.22% per patient-year. Actuarial freedom from reoperation was 99.1% at 1 and 10 years, and 85.1% at 15 years.

**Conclusion:**

Mechanical valve replacement in West African children has excellent outcomes in terms of mortality, valve-related events, and reoperation rate. Preoperative left ventricular dysfunction is the primary determinant of mortality within the first 2 years of valve replacement. The risk of valve-related complications is acceptably low. Anticoagulation is well tolerated with a very low risk of bleeding even in this socioeconomic setting.

## Background

The West African sub-region has poor health infrastructure similar to most of the developing countries in Africa. Gross National Product (GNP) in Africa is less than $1800 per inhabitant compared to $24000-$31000 in North America and Western Europe [1]. The worldwide distribution of cardio-thoracic surgeons (and cardiothoracic surgery) very closely follows the distribution of GNP; Africa has only 1% of the world's population of cardiothoracic surgeons [1]. Cardiac surgery and subsequent long term postoperative management raises serious concerns. Consequently, mechanical valve replacement in Africa is undertaken with some disquiet. Mechanical valve replacement commits the patient to lifelong anticoagulation and its associated risks of bleeding and embryopathy in pregnancy. Valve-related morbidity such as prosthetic valve thrombosis, endocarditis, and non-structural dysfunction are additional concerns that may require reoperation. Reoperation in developing countries is often an insurmountable economic hurdle for most families.

The present study was prompted by the lack of data on the outcome of mechanical valve replacement in children from the West African sub-region. We report early and late outcomes of mechanical valve replacement of the left heart in West African children over a 15-year period.

## Methods

### Study design

We retrospectively reviewed results of consecutive children undergoing mechanical valve replacement of the left heart in the study period. Hospital records of the selected patients were obtained for the purpose of the review.

### Study setting

Established in 1989, Ghana's National Cardiothoracic Center is a referral center and the only tertiary institution in the country for cardiothoracic pathology. It also serves as a cardiothoracic referral base for many of the West African countries where cardiothoracic surgery is not actively practiced due to lack of facilities.

### Patients

Between January 1993 and December 2008, 114 consecutive patients of age ≤18 years underwent left heart mechanical valve replacement at our institution. Operation records and patients' case notes were retrospectively reviewed. The study end-points included mortality, valve-related events, and reoperation. Valve-related events studied were prosthetic valve thrombosis, thromboembolism, prosthetic valve endocarditis, nonstructural dysfunction, and major bleeding events.

Procedures performed include mitral valve replacement (MVR), aortic valve replacement (AVR), or mitral and aortic valve replacements (MAVR) using mechanical prostheses.

### Operative Technique

In all patients median sternotomy was used to establish full cardiopulmonary bypass and moderate systemic hypothermia. Myocardial protection was employed by infusion of cold crystalloid cardioplegia (St. Thomas' Hospital solution) through the root or coronary ostia of the cross-clamped ascending aorta. This was repeated every 20-25 minutes and augmented by the use of topical hypothermia with saline at 4°C.

The mitral valve was approached through the inter-atrial septum in most cases. The aortic valve was approached through a standard aortotomy. The type of valve implanted is shown in table 1. Implanted valve sizes ranged from 27-31 for MVR and 19-23 for AVR.

**Table 1:** Implanted Valves.

VALVE	Number
Bileaflet mechanical	
Sorin (Sorin Biomedica, Sallugia, Italy)	97
St. Jude Medical (St. Jude Medical; St. Paul, MN)	11
Monoleaflet mechanical	
Sorin (Sorin Biomedica, Sallugia, Italy)	6

The modified De Vega annuloplasty technique [2] was used for repair of non-structural tricuspid regurgitation (TR).

### Anticoagulation protocol

Post-operative anticoagulation is initiated with unfractionated heparin as a continuous infusion (300units/kg/day) adjusted to an activated partial thromboplastin time of twice the control value after postoperative bleeding has come under control, usually on post-operative day (POD) one. Oral warfarin is begun concomitantly on the 2^nd ^POD. Heparin infusion is discontinued when the target international normalized ratio (INR) is attained. The target INR was 2.0-3.0 for AVR, and 2.5-3.5 for MVR and MAVR. The INR is adjusted upward by 0.5 units in the presence of atrial fibrillation or left ventricular dilatation/dysfunction. In our earlier experience (before 2001), a target INR of 2.5-3.0 was used for all patients.

### Discharge and Follow up

Follow up consists of clinical evaluation, anticoagulation control, prophylaxis for rheumatic fever, and antibiotic cover for endocarditis-prone procedures. Transthoracic echocardiogram is performed at least once a year.

Patients were usually discharged with an INR in the target range and followed up on a patient-specific 4-12 week interval at our institution's out-patient clinics. Non-Ghanaian West African patients were followed up 3 monthly at the Center and by telephone contact after INR testing using local laboratory facilities.

We emphasize patient/parental education before discharge. This effort is directed by a clinical pharmacist-led team. We focus attention on the need for regular monitoring and control of anticoagulation, food and drug interactions with warfarin, and prophylaxis for both endocarditis and rheumatic fever. We generally require patients to report for follow up every 4 weeks. Patients living beyond 100 km of our institution (including our non-Ghanaian West African patients) may be seen once every 8-12 weeks. For patients with such proximity problems, telephone consultation regarding dosage adjustment for warfarin and advice on endocarditis prophylaxis is an important adjunct to the clinical follow up.

### Statistical Analysis

Statistical analyses were performed using SPSS 16.0 software. Continuous variables are expressed as mean ± standard deviation. Actuarial curves were computed using the Kaplan-Meier survival analysis technique. The incidence of multiple events in individual patients is reported as a linearized event rate. Morbidity and mortality reporting are in keeping with the guidelines proposed by the 2008 ad hoc Liaison Committee for standardizing definitions of prosthetic valve morbidity and mortality [3].

## Results

The 114 patients who qualified for inclusion showed a female preponderance of 57.9%; they included 13 (11.4%) non-Ghanaian West Africans. Their ages ranged from 6-18 years (13.3 ± 3.1 years). Seventy-nine (69.3%) patients were in NYHA Class III; the remaining patients were in NYHA Class IV. Advanced cardiac disability (reflected in the high NYHA class) was attributable to prolonged illness without appropriate treatment. In most cases, financial constraint was responsible for the delay in seeking treatment resulting in progressive cardiac dysfunction. Even after diagnosis and recommendation for surgery, less than 20% could afford surgery within one year of diagnosis.

The nutritional status of the patients was acceptable except for some patients in NYHA IV who had preoperative cardiac cachexia and muscle wasting most evident after diuresis as part of their medical optimization for surgery. For hospital survivors, preoperative nutritional depletion sometimes manifested as prolonged mechanical ventilation (extra 4-7 days) and Intensive Care Unit stay. Nutritional status however improved promptly with restoration of cardiac function and enteral feeding after surgery.

Complete follow up was available for 108 patients (94.7% complete). Follow up ranged from 0.2-15.9 years (median 9.1 years).

The etiology of valve pathology (Table 2) was rheumatic in 104 (91.2%) patients. In rheumatic valve disease, valvar regurgitation was the dominant hemodynamic abnormality in both isolated and double valve involvement (Table 3).

**Table 2:** Etiology of valve pathology.

Etiology	Number	Percentage
Rheumatic	104	91.2
VSD	4	3.5
Endocarditis (Aortic)	2	1.7
SAS + Valvar AS	1	0.9
MV prolapse (Marfan's)	1	0.9
PVT	1	0.9
Annulo-aortic ectasia	1	0.9

**AS **- aortic stenosis; **MV **- mitral valve; **PVT **- prosthetic valve thrombosis; **SAS **- subaortic stenosis; **VSD **- ventricular septal defect.

**Table 3:** Valvar hemodynamics in rheumatic heart disease.

	Regurgitation	Stenosis	Total
Isolated mitral valve involvement	83 (96.6%)	4 (3.4%)	87
Isolated aortic valve involvement	7	0	7
Double valve pathology	10	0	10

MVR was performed in 91 (79.8%) patients, AVR in 13 (11.4%) and MAVR in 10 (8.8%) patients.

Four patients required AVR at the time of ventricular septal defect (VSD) closure; the VSD was associated with aortic cusp prolapse and irreparable degeneration in these patients. Three of the four patients presented late in their teens having suffered previous endocarditis resulting in deformed, unsalvageable aortic valve leaflets. The fourth patient had a bicuspid valve with shallow sinuses; a durable repair was not deemed feasible. Preoperative echocardiography and right heart catheterization excluded hypertensive pulmonary vascular disease in these patients (ages 9, 17, 17, and 18 years).

Tricuspid valve annuloplasty using the modified De Vega technique was performed concomitantly in 45 (39.5%) cases. Forty-one of these (91%) were associated with isolated mitral valve disease and 4 (9%) were due to mitral and aortic valve pathology. None was associated with isolated aortic valve pathology.

On echocardiographic follow up, there was one documented case of moderate tricuspid regurgitation in the patients who had undergone tricuspid valve repair using the modified De Vega annuloplasty. This patient had rheumatic mitral regurgitation and non-structural TR with poor pre-operative left ventricular function (ejection fraction of 42%). LV dysfunction persisted postoperatively with evolution into dilated cardiomyopathy and moderate TR despite successful MVR. Tricuspid regurgitation was mild or absent in the remainder of the patients. We have had acceptable results with the modified De Vega technique in a prior experience [4].

Preoperative left ventricular dysfunction (ejection fraction < 45%) was the most important factor contributing to both early and late mortality; it was present in 5 of 6 (83%) early deaths and in 3 of 6 (50%) late deaths. Most survivors, however, demonstrated improvement in LV dysfunction on long-term follow up. Two patients (including the one with moderate TR) showed persistent LV dysfunction and dilated cardiomyopathy with atrial fibrillation after 2 and 3 years of follow up respectively. They are on appropriate decongestive medication and antiarrhythmic drug therapy.

### Mortality

The overall 30-day mortality was 5.3% (6 patients, Table 4); late mortality occurred in 6 patients (Table 5) at a linearized rate of 0.67% per patient-year. The actuarial survival was 98.1% at 1 year, 97.0% at 5 years, and 94.0% at 10 and 15 years.

**Table 4:** Early mortality.

Causes of early death	Number	Comment
Postoperative low cardiac output	4	Preoperative LV dysfunction (EF<45%).
Cerebral re-infarction	1	Preoperative cerebral embolism.
Prosthetic valve endocarditis and CHF	1	Preoperative endocarditis and CHF.

**CHF **- congestive heart failure, **EF **- ejection fraction.

**Table 5:** Late mortality.

Late mortality	Number	Comment
Prosthetic valve thrombosis	2	Died 9 and 14 years respectively after MVR.
		Non-compliance with follow-up.
Progressive LV dysfunction postoperatively	3	All died within 2 years of valve replacement.
Sudden cardiac death	1	Progressive rheumatic aortic valve regurgitation post-MVR. Parents declined reoperation.

**LV **- left ventricular.

### Valve-related events

#### 1) Prosthetic Valve Thrombosis (PVT) and thromboembolism

PVT occurred in 5 patients (Table 6) at 0.56% per patient-year. Four occurred in the mitral position and one in the aortic position. Thrombosis was responsible for all four prosthetic mitral valve obstructions. In the fifth patient, coexistent thrombus and pannus caused aortic valve obstruction. Only two of the four mitral PVTs survived. Of the two survivors, thrombolysis with streptokinase was successful in one; the other required reoperation to replace the thrombosed valve.

**Table 6:** Development of PVT.

Patient	Surgery	INR at PVT diagnosis	Mechanism of PVT	Surgery to PVT Interval (years)
**1**	MVR	2.1	Thrombus	0.3
**2**	MVR	?	Thrombus	0.5
**3**	AVR	2.0	Thrombus and Pannus	9.1
**4**	MVR	INR not done for > 8 months	Thrombus	9.0
**5**	MVR	INR not done for > 18 months	Thrombus	15.0

Time from surgery to PVT: Mitral - 6.2 ± 7.3 years; All PVT cases - 6.8 ± 6.3 years. AVR - aortic valve replacement, MVR - mitral valve replacement, PVT - prosthetic valve thrombosis.

The only aortic PVT in the series responded partially to thrombolysis; the obstruction was due to a combination of thrombus and pannus. She underwent elective replacement of the aortic prosthesis.

Actuarial freedom from PVT was 98.7% at 1 and 5 years, 96.9% and 94.0% at 10 and 15 years respectively.

#### 2) Thromboembolism

There were no documented postoperative embolic events in this series.

#### 3) Prosthetic Valve Endocarditis (PVE)

Early PVE occurred in one patient (0.9%) and was contributory to the patient's death. This was a six year old boy who had left ventricular failure from rheumatic mitral incompetence complicated by preoperative bacterial endocarditis. Failing medical management, MVR was arranged as an urgent measure. Postoperatively, he remained ill in a low cardiac output state until his demise on the 16^th ^POD. The postmortem examination showed peri-prosthetic micro-abscesses.

#### 4) Major Bleeding Event

There was 1 (0.9%) major bleeding event in this series. Severe upper gastrointestinal bleeding occurred in a 14 year-old boy nine days after MVR when the INR was 2.0. He required a laparotomy the next day when conservative measures failed. A bleeding ulcer in the first part of the duodenum was found and hemostasis was secured by suture.

### Reoperation

**Figure 1:**
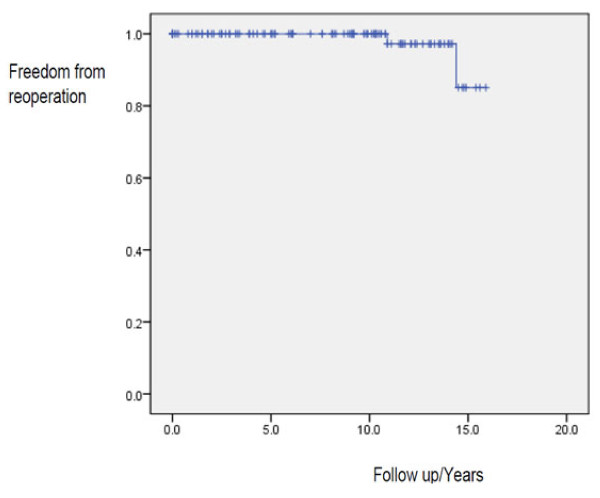
**Actuarial freedom from reoperation**. Kaplan-Meier estimates of freedom from reoperation after 15 years' follow up showing actuarial figures of 99.1% and 85.1% at 10 and 15 years respectively.

Reoperation was necessary in 2 patients (0.22% per patient-year). The actuarial freedom from reoperation (Figure 1) was 99.1% at 1, 5, and 10 years and 85.1% at 15 years.

The first reoperation was for PVT in an 8 year-old boy 6 weeks after MVR with a Sorin monoleaflet valve (Sorin Biomedica, Sallugia, Italy). The INR at the time of PVT diagnosis was 2.0. The thrombosed valve was urgently replaced with a bileaflet valve from the same manufacturer. The second reoperation was for another PVT that was only partly responsive to thrombolytic therapy after 11 years of AVR.

## Discussion

Rheumatic heart disease was responsible for more than 90% of patients undergoing mechanical valve replacement in the present study. Late presentation of patients for surgery was characteristic. Advanced rheumatic valve pathology made valve replacement the preferred option. Cardiologists may delay the recommendation of valve replacement in children until greater clinical disability and failed medical therapy occur. This practice has been based on the belief that children tolerate anticoagulation poorly and that valve-related complications are far too common in children. We have shown in this study the excellent tolerability of anticoagulation in West African children and the acceptable valve-related morbidity. In addition, our results demonstrate that delay in the institution of surgical treatment allows progressive deterioration of ventricular function and contributes primarily to both early and late mortality.

### Mortality

Preoperative left ventricular dysfunction was the primary contributor to both early and late mortality. Patients with preoperative left ventricular dysfunction experienced early postoperative low cardiac output syndrome or relentlessly progressive cardiomyopathy of mitral incompetence after discharge from hospital. In developing countries, rheumatic heart valves tend to deteriorate rapidly due to repeated episodes of acute rheumatic carditis that lead to severe debilitating disease, ventricular dysfunction, and premature death [5,6]. Such advanced disease allows progressive deterioration of LV function even after valve replacement. Delay in seeking treatment as a result of inadequate health infrastructure and poor health care financing contributes to creating a pool of children who present with preoperative LV dysfunction. In Ghana, state medical insurance is available for registered citizens but gives limited coverage for primary health care, anticoagulation testing, and medications (including warfarin and generic decongestive drugs for heart failure). There is no health insurance cover for open heart surgery. The Ghana Heart Foundation (GHF), a non-governmental organization set up by the senior author (KF-B) at the inception of our institution assists Ghanaian patients to the tune of 50% of the cost of open heart surgery. In dire emergencies, the GHF may bear the full cost. Unfortunately, if the patient is unable to afford the cost of surgery, appeals to the public and philanthropists for financial assistance becomes the only option with sometimes unfortunate consequences. This situation, common in much of West Africa, contributes to the situation where children are kept waiting until severe preoperative LV dysfunction occurs before presentation for surgery. As our results show, preoperative LV dysfunction may progress relentlessly even after successful valve replacement. Workers from other developing countries have reported similar outcomes attributable to late presentation of rheumatic heart disease and poor preoperative ventricular function [7,8], with comparable early mortality. This underscores the importance of early intervention in rheumatic heart disease before irreversible deterioration of ventricular function occurs.

The presence of active rheumatic carditis (suspected in 2 early deaths) and preoperative infective endocarditis (confirmed in 1 early death) were additional factors contributing to early mortality.

Late death occurred in 6 (5.3%) patients at a linearized rate of 0.67% per patient-year. The actuarial survival at 1 and 15 years were 98.1% and 94.0% respectively. WHO figures from 2008 suggest that the 15-year life expectancy of adolescent male and female Ghanaians with median age similar to the study group (13 years) is about 96.5% [9]. Mechanical valve replacement is thus associated with a near-normal 15-year life expectancy in these patients.

In a similar patient cohort, Akhtar and colleagues [7] reported 11 (12.4%) late deaths; patient survival at 1 and 10 years was 87.5% and 82.9% respectively. Barnard and colleagues [8] recently reported 14.3% late death with actuarial survival at 5 years of 80%. These workers [7,8] confirm the results of the current study that the risk of on-going death for hospital survivors of valve replacement for rheumatic heart disease can be significant. This may be attributed to a combination of progressive deterioration of ventricular function in late presenters and poor anticoagulation control.

Compared to these reports [7,8], valve-related mortality was relatively low in this study (3 of 114, or 2.7%). This is probably a reflection of higher compliance with postoperative management protocols. Aktar and colleagues [7] reported that most of their patients suffering fatal events had erratic follow-up with sub-therapeutic anticoagulation. Because of the formidable financial hurdle of 'out-of-pocket' financing of open heart procedures in West Africa, most patients and parents have a high motivation to avoid reoperation. With appropriate guidance most tend to comply with follow-up management protocols. Other factors may be operative in the lower valve-related mortality in the present study. The use of 3^rd ^generation bileaflet mechanical prostheses with lower thrombogenicity in the majority of our patients may be contributory. Additionally, out-patient follow-up by our team of surgeons and clinical pharmacists using patient-specific anticoagulation regimens probably played a role as well [10].

The impact of age and underlying etiology for mechanical valve replacement must not be overlooked. The report of Ackermann and coworkers [11] demonstrate that replacement of the systemic atrioventricular valve with a mechanical prosthesis in children aged less than 6 years in whom the underlying etiology is non-rheumatic may portend a somewhat higher mortality. They showed a survival of 73% at 1 year and 65% at 5, 10, and 15 years. The youngest patient in our study was 6 years of age; rheumatic heart disease was the underlying etiology in close to 91% of our cases. Outcomes for mechanical valve replacement of the left heart in children therefore are dependent on the etiology and age of the patient cohort.

### PVT and thromboembolism

PVT and thromboembolism after mechanical valve replacement are both related to the balance between thrombogenicity and the adequacy of anticoagulation. Two forms of PVT are usually described - obstructive and non-obstructive [12].

Non-obstructive PVT commonly occurs in the highly unstable early postoperative period [12]. It causes minimal local symptoms but predisposes the patient to systemic thromboembolic phenomena at a rate of 0.7-6% per patient-year [13]. From the developing world, John and colleagues [5] reported a thromboembolic rate of 2.8% or 0.8% per patient-year. In a subsequent report, their thromboembolic rate dropped to 0.41% per patient-year for MVR [14]. During the first six postoperative months, the thromboembolic risk is up to seven times greater than in the months and years afterward. After AVR, thromboembolic risk falls from 16% per patient-year in the early postoperative period to 1.4% per patient-year at 5 years. Similarly, after MVR, the risk falls from 21% per patient-year to 2.5% per patient-year [15]. We did not document any episode of thromboembolism in our study.

Unlike non-obstructive PVT, obstructive PVT is an acute life-threatening complication demanding urgent intervention. The incidence of obstructive PVT for mechanical valves varies between 0.3-1.3 percent per patient-year [13]; our experience compares favorably. The first postoperative year is marked by a 24% incidence of obstructive thrombosis, with a stable incidence between the second to fourth years of approximately 15%, and a subsequent decrease thereafter [16]. Poor health infrastructure in most developing nations would predict a higher rate of obstructive and non-obstructive PVT but our results and those of others [5,14] indicate that this may be unfounded.

Among the commonest precipitating factors for mechanical valve thrombosis are inadequate anticoagulation and poor patient compliance. In this study, obstructive PVT (4.4%) occurred at a linearized rate of 0.56% per patient-year with an overall mean time to development of 6.8 ± 6.3 years (Table 6). The incidence of acute thrombotic occlusion of a mechanical replacement device has been reported to average 0.03% to 8% per patient-year [17,18]. From the Montreal Heart Institute, Durrleman and coworkers [17] found the time interval from first valve replacement to prosthetic valve thrombosis was 3.25 ± 3.50 years. At presentation of PVT, the INR was less than 2.5 in 54% of their patients, with inadequate anticoagulation management in 26% and poor compliance in another 26%. Two of our patients who developed obstructive PVT were notoriously non-compliant for several years without apparent adverse effects. Incidentally, these were the only two who suffered late death from PVT after 9 and 15 years of MVR. Patient-related factors therefore heavily influence the occurrence of PVT and high patient motivation to comply with postoperative anticoagulation management is a sine qua non to improved long-term outcome.

### Reoperation

In terms of freedom from reoperation, mechanical valve replacement for rheumatic heart disease is superior to valve repair. A reoperation rate of 23% after valve repair for rheumatic heart disease has been reported [19,20] with actuarial freedom from reoperation of 78% at 5 years, 65% at 10 years, and 49% at 15 years [20]. Freedom from moderate or severe mitral regurgitation may be as low as 32 ± 3.9% 10 years after valve repair for rheumatic heart disease [21]. In our experience, reoperation occurred at 0.22% per patient-year with freedom from reoperation of 99.1% and 85.1% at 10 and 15 years respectively (Figure 1).

The financial implication of reoperation in our population is considerable. In 2005, 47 children (≤15 years) were diagnosed by echocardiography as rheumatic heart surgical candidates at our institution. Of this number, only 7 (14.9%) underwent surgical treatment that same year. Lack of funding was the primary reason for the delay. Where health financing is limited, a major financial hurdle is imposed by the necessity of reoperation for a failed valve repair, most of which is dependent on progression of the underlying rheumatic cardiac pathology and not on surgical technique [22]. Mechanical valve replacement in this setting confers a significant advantage in populations where funding for open-heart procedures is deficient. The drawback to this management strategy is the impact and consequences of lifelong anticoagulation.

The need for reoperation resulting from patient-prosthesis mismatch in growing children was not realized in our experience. The presence of gross cardiomegaly in most of our patients, all of whom presented with long-standing disease in NYHA class III or IV, allowed the implantation of adult-sized prostheses which ostensibly curtailed the development of this complication.

### PVE

PVE occurred at 0.11% per patient-year and was responsible for one early death in this series. This was of the culture-negative variety detected pre-operatively in a six year-old boy. Mechanical prostheses predispose to device-related infections especially those caused by coagulase-negative staphylococci, which are able to adhere to a variety of surfaces and produce an antibiotic-resistant biofilm [23,24]. The risk for early PVE is higher in patients undergoing valve replacement surgery during active infective endocarditis, especially if the causal organism is unknown. Once established, PVE carries a mortality rate that may be as high as 70% [25]. The established treatment for PVE is rigorous intravenous antimicrobial therapy, although this has extremely limited success. The majority of cases require surgical removal and replacement of the infected prosthesis. Unfortunately, in our patient this could not be carried out before he succumbed.

### Major Bleeding Event

After mechanical valve replacement, major bleeding and thromboembolic complications are notably commoner in patients with a high variability in anticoagulation control [26]. Major bleeding events occur in 2.4-4.6% per patient-year after warfarin anticoagulation for mechanical valve replacements [27]. We believe the low rate of major bleeding events in our study (0.11% per patient-year) testifies to the excellent tolerability of warfarin in our pediatric population. Our experience is similar to other workers from developing countries [14].

### Prospects for Selected Patients

This patient cohort included two categories of notable patients. First is the female with child-bearing potential. Four in the group have successfully borne children without complications. Our anticoagulant protocol involves substitution of warfarin with subcutaneous unfractionated heparin in the first trimester and at elective Caesarian section through a collaborative effort with the attending obstetricians. Three were delivered by elective Caesarean section at 37 weeks gestation. The fourth had a spontaneous vaginal term delivery at a peripheral hospital without complications. We did not encounter warfarin embryopathy or fetal wastage in this patient cohort.

The second category consists of two patients who have homozygous (SS genotype) sickle cell hemoglobinopathy. Notably, the frequency of sickling crises has drastically reduced in both patients since undergoing mechanical valve replacement with institution of anticoagulation. Some workers have previously pointed out that warfarin may protect against sickling crises [28,29].

### Study limitations

Inherent limitations in all retrospective analysis apply to this study. Because complete follow-up was not available for 6 patients, the possibility of missed events must be kept in mind in the interpretation of the results. Differences in socio-cultural behavior of different populations with regard to compliance with post-operative management protocols impact on the generalizability of the study results.

## Conclusion

Mechanical valve replacement in West African children has excellent outcomes in terms of early and late mortality, valve-related events, and reoperation rate.

Preoperative left ventricular dysfunction is the primary determinant of mortality within the first 2 years of valve replacement.

Anticoagulation is well tolerated with a very low risk of bleeding.

The risk of valve-related complications is acceptably low even in this socioeconomic setting.

Efforts at improving long-term surgical outcomes for mechanical valve replacement should focus on early surgical intervention and improved control of anticoagulation for mechanical valves.

## Abbreviations

AS: aortic stenosis; AVR: aortic valve replacement; EF: ejection fraction; GNP: gross national product; INR: international normalized ratio; LV: left ventricle; MAVR: mitral and aortic valve replacements; MV: mitral valve; MVR: mitral valve replacement; NYHA: New York Heart Association; POD: postoperative day; PVT: prosthetic valve thrombosis; SAS: subaortic stenosis; TR: tricuspid regurgitation; VSD: ventricular septal defect.

## Competing interests

The authors declare that they have no competing interests.

## Authors' contributions

FE conceived the study, performed literature search, collected data, performed statistical analysis, and drafted the manuscript. EA contributed to the data analysis, editing and reviewing the manuscript. MMT contributed to the study design, editing and reviewing the manuscript. MT participated in data collection and editing of the manuscript. KF-B provided study background information, supervised the work, and reviewed the manuscript. All authors read and approved the final manuscript.
